# Negative effects of mindfulness-based cognitive therapy and cognitive behavioural analysis system of psychotherapy for patients with ‘difficult to treat’ depression: protocol for a systematic review and individual participant data meta-analysis

**DOI:** 10.1136/bmjopen-2025-106362

**Published:** 2026-04-16

**Authors:** Maria Niemi, Johannes Michalak, Maria Velana, Thorsten Barnhofer, Mathias Harrer

**Affiliations:** 1 Department of Global Public Health, Karolinska Institutet, Stockholm, Sweden; 2 Department of Psychology and Psychotherapy, Witten/Herdecke University, Witten, Germany; 3 University of Surrey, Guildford, UK; 4 Department of Clinical, Neuro- and Developmental Psychology, WHO Collaborating Centre for Research and Dissemination of Psychological Interventions, Vrije Universiteit Amsterdam, Amsterdam, The Netherlands

**Keywords:** Meta-Analysis, Adverse events, Depression & mood disorders, Mindfulness

## Abstract

**Abstract:**

**Introduction:**

Mindfulness-based interventions are widely used, yet concerns about potential negative effects—particularly those related to mindfulness meditation practice—have gained increasing attention. Individuals with difficult-to-treat depression (DTD) represent a population of particular relevance due to heightened vulnerability, but comparative evidence on clinically relevant negative outcomes of mindfulness-based cognitive therapy (MBCT) versus established alternative psychotherapies in this group is lacking. This protocol describes a systematic review and individual participant data (IPD) network meta-analysis to assess and compare the incidence of clinically relevant negative outcomes associated with MBCT and the cognitive behavioural analysis system of psychotherapy (CBASP), an established individual psychotherapy for DTD.

**Methods and analysis:**

Randomised controlled trials of MBCT and CBASP for adults with DTD were identified through systematic searches of major databases. Eligible studies must compare MBCT or CBASP (alone or with treatment as usual) to each other or to control groups. The primary outcome is clinically significant deterioration, defined as a ≥6-point increase on the Patient Health Questionnaire-9 or equivalent. Secondary outcomes are suicidality and treatment dropout. IPD will be requested from trial investigators; aggregate data will be used when IPD is unavailable. One-stage random-effects IPD network meta-analyses will be conducted to integrate direct and indirect evidence and to examine participant-level moderators of deterioration. Adverse events reported in the included trials will be summarised descriptively at the study level.

**Ethics and dissemination:**

No local ethical review was required following consultation with the Swedish Ethical Review Authority. Primary trial investigators obtained local ethical approval and will share pseudonymised IPD. Findings will inform clinical decision-making and guideline development by strengthening the evidence base on potential negative effects of MBCT and CBASP in adults with DTD, including identification of subgroups at increased risk. Results will be disseminated through peer-reviewed publication and accessible summaries for relevant stakeholders.

**PROSPERO registration number:**

CRD42022332039

**STRENGTHS AND LIMITATIONS OF THIS STUDY**

- Leveraging an individual participant data meta-analysis allows for more precise assessment of negative effects and enables detailed moderator analyses that are not possible with aggregate data approaches.
- The use of a network meta-analytic framework enables integration of direct and indirect evidence across trials comparing mindfulness-based cognitive therapy, cognitive behavioural analysis system of psychotherapy and control conditions, allowing estimation of comparative effects even where head-to-head trials are limited.
- Standardising outcome measures across studies using a common metric enhances comparability but may result in some loss of information or sensitivity to subtle differences in negative outcomes.
- Differences in the availability and reporting of individual-level variables across studies may limit the ability to fully explore all potential moderators of negative effects, and reliance on data sharing from original authors could restrict the inclusion of eligible studies.

## Introduction

 Mindfulness-based interventions (MBIs) have been extensively studied across a wide range of clinical conditions and populations. A recent umbrella review synthesising evidence from 44 meta-analyses demonstrated that MBIs are broadly efficacious, showing significant benefits across multiple mental health outcomes.^1^ Among these interventions, mindfulness-based cognitive therapy (MBCT)^2^ was specifically developed for the treatment of depression and has been shown to be effective not only for relapse prevention in recurrent depression,^3^ but also for acute and persistent depressive symptoms.^4 5^

Despite well-established efficacy, increasing attention has been directed toward potential negative effects of MBIs, particularly those related to mindfulness meditation practice. Reports from qualitative studies, surveys and clinical investigations indicate that meditation-related adverse experiences—ranging from transient distress to functionally impairing and persistent symptoms—are not uncommon.^6^,^8^

To date, however, conclusions regarding the relative safety of MBCT remain limited. Claims that adverse effect rates in MBCT are comparable to those of other evidence-based psychotherapies are largely based on indirect comparisons across heterogeneous studies using different assessment methods.^9^,^11^

Many psychotherapy trials do not systematically assess adverse events (AEs), and definitions and measurement strategies vary substantially, limiting comparability across treatments.^12 13^ This limitation is particularly pronounced for MBIs, as meditation-specific adverse experiences are not routinely or systematically assessed in randomised controlled trials of MBCT.

Consequently, comparative evaluations of safety across interventions are constrained and must rely on clinically relevant outcomes that can be consistently derived from existing randomised controlled trial datasets. In this context, symptom deterioration and suicidality represent particularly important and measurable indicators of negative effects that can be derived from existing randomised controlled trial datasets.^14^ Treatment dropout constitutes an additional indicator of treatment acceptability and potential burden and may reflect negative experiences or tolerability problems during treatment.^15^

Individuals with difficult-to-treat depression (DTD)—a broader and clinically meaningful construct encompassing persistent, impairing depressive courses regardless of strict treatment resistance criteria^16^,^18^—represent a population of particular relevance for the study of negative effects. DTD is associated with elevated vulnerability, including higher rates of early life adversity and suicidality,^19^ factors that have also been linked to increased risk of adverse experiences during psychological interventions.^20^

In order to study the negative outcomes of MBIs, it is therefore timely to compare these to other established treatment methods among this patient population. The cognitive behavioural analysis system of psychotherapy (CBASP)^21^ is an empirically supported treatment specifically developed for individuals with persistent depression and has been evaluated in randomised controlled trials targeting this population.^22 23^ Because CBASP is the most-studied intervention for DTD (ie, persistent depression), it represents a clinically meaningful and appropriate comparator for MBCT in patients with DTD.

Notably, while both interventions are applied in populations characterised by persistent depressive symptoms, they differ fundamentally in their therapeutic focus and proposed mechanisms of change. CBASP emphasises interpersonal learning and perceived interpersonal consequences, whereas MBCT primarily targets decentring from maladaptive cognitive and emotional processes through mindfulness practice. Moreover, MBCT is typically delivered in a group format, whereas CBASP is most commonly delivered as an individual, one-to-one psychotherapy.

Direct head-to-head comparisons between MBCT and CBASP are limited, and most available trials compare each intervention separately with treatment-as-usual or other control conditions. Consequently, there is a lack of comprehensive comparative evidence regarding the relative safety and acceptability of these interventions in adults with DTD.

Moreover, data on negative effects are often insufficiently reported in published trials, with substantial heterogeneity in definitions, measurement and reporting formats,^12^ limiting the possibility of meaningful comparison at the aggregate level.

To address these gaps, the present study will conduct a systematic review and individual participant data (IPD) network meta-analysis of randomised controlled trials evaluating MBCT and CBASP in adults with DTD. This approach enables integration of direct and indirect evidence across interventions and control conditions, while allowing harmonised outcome definitions and examination of participant-level moderators of risk.

The primary objective of the present study is to compare rates of negative effects, operationalised as symptom deterioration, suicidality and dropout. A further objective is the identification of participant-level predictors of negative effects across both treatments. Moreover, AEs reported in the included randomised controlled trials will be summarised descriptively at the study level. Given the absence of prior direct comparative evidence, all comparative analyses were specified as exploratory and non-directional.

More specifically, the objectives of the present study will be as follows:

1. To compare rates of clinically significant symptom deterioration between MBCT and CBASP in patients with DTD using an IPD network meta-analytic framework (primary outcome).
2. To compare levels of suicidality between MBCT and CBASP in patients with DTD within the network meta-analysis.
3. To compare rates of treatment dropout between MBCT and CBASP in patients with DTD as an indicator of acceptability and tolerability within the network meta-analysis.
4. To summarise rates of AEs reported in trials of MBCT and CBASP in patients with DTD.
5. To investigate study- and patient-level moderators of clinically significant symptom deterioration.

## Methods

### Study design

This IPD meta-analysis will include any randomised controlled study investigating the efficacy of MBCT/CBASP alone or adjunct to TAU (see Comparator section) for reducing symptoms of depression in adults with DTD.

The analytic focus is on MBCT and CBASP as the primary interventions of interest, enabling a clinically interpretable comparison of safety and acceptability within a relatively homogeneous population and intervention context. Control conditions are incorporated as comparators within the network to allow estimation of relative effects between MBCT and CBASP through integration of direct and indirect evidence.

We will assess rates of clinically significant deterioration, suicidality, dropout and AEs in MBCT and CBASP for DTD. This IPD meta-analysis is prospectively registered in PROSPERO, and any key changes or amendments will be documented there. The Preferred Reporting Items for Systematic Reviews and Meta-Analyses IPD statement will be followed for the reporting of this study.

The study was initiated in February 2023, including study planning and preparatory work as well as the initiation of IPD requests. The systematic literature search was conducted up to 30 May 2025, in accordance with the registered protocol, and the study is expected to be completed by the end of 2026. The search strategy allows for updates prior to the final analyses if required. The anticipated completion date will be updated in the PROSPERO record to reflect the revised timeline, with study completion expected by the end of 2026.

### Participants

We will include studies recruiting adults with a current major depressive disorder as well as trials enrolling mixed samples with either residual depressive symptoms or a current depressive episode in the context of a persistent, chronic or difficult-to-treat course. In trials including such mixed samples, participants presenting with residual symptoms only will be excluded from the primary one-stage IPD analyses. As DTD does not rely on a single standardised operational definition, we will apply a broad and inclusive approach and include studies that defined eligibility based on prior non-response or non-remission, treatment resistance and/or chronicity. Studies including participants younger than 18 years, and studies exclusively recruiting participants older than 65 years, will be excluded.

#### Interventions

The interventions of interest are MBCT and CBASP, delivered either as stand-alone treatments or as adjuncts to treatment as usual (TAU). MBCT will be defined as a manualised intervention based on the original protocol developed by Segal *et al*.^2^ Given that MBCT was originally designed for relapse prevention, trials that introduce minor, well-described adaptations to accommodate participants with persistent or current depressive symptoms (eg, modified psychoeducation, pacing or practice emphasis) will be eligible, provided that the core components of the MBCT programme are retained.

CBASP will be defined as a manualised psychotherapy developed specifically for individuals with chronic or persistent depression.^21^ CBASP integrates behavioural, cognitive and interpersonal strategies, with a primary focus on perceived interpersonal consequences and interpersonal learning, and is typically delivered in an individual, one-to-one format.

#### Comparator(s) or control(s)

Comparator conditions will include TAU and other control conditions (eg, waitlist, minimal intervention or psychoeducation) used in eligible randomised controlled trials. These comparators will be incorporated as nodes within the evidence network to enable estimation of relative effects between MBCT and CBASP through integration of direct and indirect evidence.

Trials directly comparing MBCT and CBASP, as well as trials comparing either intervention with TAU or other control conditions, will be eligible for inclusion. The primary analytic focus is on comparative safety and acceptability of MBCT and CBASP; control conditions serve as common comparators within the network rather than as interventions of substantive interest in their own right.

### Outcomes

The primary outcome will be the rate of clinically significant symptom deterioration, assessed using standard depression measures, including self-report questionnaires (eg, the Patient Health Questionnaire-9 (PHQ-9) or Beck Depression Inventory) and clinician-rated instruments (eg, the Hamilton Depression Rating Scale). If multiple measures are used in a single study, preference will be given to the measure most commonly used across studies. To ensure comparability of symptom severity scores assessed with different instruments, values will be transformed to the PHQ-9 metric using a validated common metric approach.^24^ The common metric approach transforms scores obtained from different, but conceptually comparable, outcome measures onto a single standardised scale, thereby enabling direct comparison and joint analysis of outcomes across studies. If a measure is not part of the common metric, it will be converted using validated conversion tables to derive the common metric.^25^

We will create a dichotomous outcome indicating deterioration. Dichotomisation is commonly used in research on negative effects of psychotherapy^26^ because it facilitates the identification of clinically meaningful deterioration, which often represents relatively rare but clinically critical events. According to PHQ-9 conventions, deterioration will be defined as an increase of 6 or more points.^27^ A definition of deterioration using the 6-point change threshold may lead to sparse data (eg, one and ‘double-zero’ cells), which are often problematic for meta-analytic pooling.^28^ As a mitigation strategy, if zero cells occur in two or more trials, we will therefore also consider modelling deterioration as any negative change compared with baseline, so as to avoid computational problems.

Suicidality will be analysed as one of the secondary outcomes and will be harmonised across studies using a predefined hierarchical approach. When available, data on suicidality will be obtained from clinician-rated items (eg, Hamilton Depression Rating Scale, Item 3) and self-report items (eg, BDI, Item 9). If multiple measures of suicidality are used in a single study, preference will be given to the measure most commonly used across studies. Dichotomous outcomes will be derived to indicate the presence or emergence of suicidality. Participants will be classified as having suicidality if they scored ≥1 on the respective suicidality item. *Emergent suicidality* will be defined as any occurrence of suicidality among participants without indication of suicidality at baseline and *worsening of suicidality* as an increase of ≥1 point from baseline among participants who already report some level of suicidality.

We will report and compare dropout rates between arms as a distinct acceptability outcome; this will complement the handling of missing symptom data via multiple imputation.

Reporting and definitions of AEs vary substantially across psychotherapy trials and are often not assessed using standardised procedures. For this reason, AEs will not be synthesised as a quantitative outcome in the primary IPD meta-analysis or included in patient-level moderator analyses. Instead, we will extract and present, for each study, the study-specific method of AE assessment and the number and type of reported AEs (and serious AEs, where available). These data will be summarised descriptively in a dedicated table.

#### Moderators

Baseline constructs will include assessments of potential psychological moderators of adverse effects. These will include potential moderators at the level of the individual participant (eg, sociodemographic and clinical characteristics) and at the level of the study. We will include patient-level characteristics in the analyses, if they are consistently available across the datasets and, where inclusion is justified, based on evidence of possible effects on treatment response or deterioration from prior research.^29^

Individual level sociodemographic and clinical characteristics that will be investigated include age,^30^ gender,^31 32^ marital status,^32^ education,^33^ employment,^34^ childhood adversity,^20^ age at onset of depression,^35^ number of previous episodes,^36^ current episode duration,^37^ current suicidality,^38^ current antidepressant medication,^39^ past antidepressants and treatment-resistance to antidepressants,^40^ comorbid anxiety disorder,^30^ comorbid obsessive-compulsive disorder,^30^ comorbid post-traumatic stress disorder,^30^ comorbid substance abuse or dependence,^30^ comorbid eating disorder,^30^ comorbid personality disorder^30^ and level of depressive symptoms at baseline.^41^ Intervention and study characteristics will include number of treatment sessions attended, treatment completer status, type of delivery (remote vs face-to-face) and type of DTD (non-response/remission, treatment-resistance, chronic depression).

### Searches

We have searched the following databases for relevant publications: EMBASE, PubMed/MEDLINE, PsycINFO, Web of Science, Scopus and the Cochrane Controlled Trials Register (see search terms in online supplemental appendix 1). Once relevant publications were identified, their bibliographic reference list was searched for any additional relevant study. We have searched for any study published up until the day of the search, on 30 May 2025, and there was no lower time limit. We searched for studies published in English, and unpublished studies were not sought.

### Data extraction (selection and coding)

Following completion of the search strategies, titles and abstracts were screened independently by two reviewers. For records selected by at least one reviewer, the full abstract was retrieved. Disagreements regarding eligibility were resolved by consultation with a third reviewer, whose decision was final. Records kept of all titles and abstracts screened, including reasons for inclusion and exclusion, and all decisions were documented and signed by the three reviewers.

As this is an IPD meta-analysis, data will be sought, rather than extracted from publications. Corresponding authors responsible for the included studies will be contacted via email by the project leaders with an invitation to participate in the review and to share the IPD from their primary study. The standard letter will include all relevant details of the study including its purpose and main research questions, the variables of interest and will include a request to share the raw data from the study. Corresponding authors of studies will be invited to serve as collaborators to the project. If corresponding authors do not respond within 4 weeks, we will send a second email request. If this fails, a second author will be contacted. In the case of further failure to make contact, we will continue until we have contacted a maximum of three authors. If none of the authors responds, or if the authors indicate that the data are unavailable, we will list the study data as unavailable. Each contacted author will receive an initial email and one reminder. If we are unable to get IPD for a study, we will extract aggregate data (eg, on AEs) from the paper of that study. Studies for which only aggregate data are available cannot be included in patient-level regression analyses examining individual participant characteristics. We will also carefully evaluate the transitivity assumption for analyses including aggregate-only studies.

### Risk of bias (quality) assessment

Study validity was assessed independently by three raters (MV, MN, TB) using the Cochrane Risk of Bias 2 (RoB 2) tool^42 43^ in accordance with recent recommendations for the operationalisation of RoB 2 in psychotherapy trials.^44^

Each study was assessed for quality by two independent reviewers, with disagreements referred to a third reviewer, whose decision was final. A sensitivity analysis will be performed excluding studies judged to be at high risk of bias, or where risk of bias is unclear.

### Planned data synthesis

To calculate a pooled estimate of the (direct and indirect) treatment contrasts included in the obtained IPD, a one-stage IPD network meta-analysis (IPD-NMA) model will be employed, adjusting for baseline prognostic factors. The effect of moderators will be assessed by adding treatment-covariate interactions to this main IPD-NMA model on a one-by-one basis, yielding an IPD network meta-regression. In all models, covariates will be centred and scaled by cluster to facilitate convergence.

The primary outcome, symptom deterioration, will be modelled using a binomial logit link. Let *y_ik_* be the deterioration status of some participant *i* included in study *k* (with *k*=1, …, *K* studies included the meta-analysis), where *j=b* represents the baseline arm and *j=t* the intervention arm in *k*. The one-stage IPD-NMA model will then be defined as follows:

$log_{e}\left(\frac{\pi_{ijk}}{1-\pi_{ijk}}\right)=\left\{ \begin{array}{l} \alpha_{k}+\sum_{p=1}^{P}\gamma_{p}\left(X_{p,ijk}-{\overset{-}{X}}_{∙p,k}\right)\;\;\;\;:\;j=b\\ \alpha_{k}+\delta_{k}+\sum_{p=1}^{P}\gamma_{p}\left(X_{p,ijk}-{\overset{-}{X}}_{∙p,k}\right)\;\;\;:\;j\neq b \end{array}\right.$


$$y_{ijk}\sim Bern\left(\pi_{ijk}\right)$$

$$\delta_{}\sim MVN(d,\Sigma );d_{1}=0$$

$$\alpha_{k}∼t_{\nu =1}(0,10);\;\delta_{k},\gamma_{p}∼t_{\nu =1}(0,2.5)$$

where *d* represents a vector containing the effect sizes in comparison to the reference treatment implicated by our network, and with Σ being a heterogeneity variance-covariance matrix with off-diagonal elements $\rho \tau^{2}$, where ρ is set to a non-zero value to account for multiarm studies. A weakly informative Half-Normal(0,0.5) prior will be used by default square root of the heterogeneity variance, τ.^45^ Given that we expect deterioration to be fairly uncommon, to avoid issues with sparse data, weakly informative Cauchy(0,2.5) and Cauchy(0,10) will be used for all slopes and intercepts, respectively.^46^ Analyses with flat N(0,100^2^) priors will be conducted as a sensitivity analysis if feasible.

The presence of network consistency will be explored by several approaches. First, we will generate graphical summaries of baseline indicators within the IPD (eg, box-whisker plots grouped by comparison). This will be used to identify potentially systematic divergences in the baseline risk across comparisons within the network, which can be the cause of inconsistency. Quantitatively, the transitivity assumption will be assessed by (1) calculating the agreement between direct and indirect evidence for comparisons in an inconsistency model including an additional inconsistency parameter *w* and (2) running a two-stage model using the ‘net split’ approach.^47^ As a sensitivity analysis, we will also (1) perform pairwise one-stage IPD meta-analyses using the direct evidence in the network and (2) employ two-stage IPD-NMA models additionally.

All models will be fitted within a Bayesian framework using Gibbs sampling as implemented in JAGS (Just Another Gibbs Sampler).^48^ The potential scale reduction factor ($\hat{R}$) will be used to assess convergence. Missing data will be handled using multilevel multiple imputation with *m*=50 sets. All models will be fitted within multiply imputed data, and parameter estimates will be aggregated by mixing the posterior draws. Multiple imputation operates under the missing at random assumption. As a sensitivity analysis, we will therefore also rerun the main model under the ‘worst-case’ scenario that all individuals who were lost to follow-up experienced reliable symptom deterioration.

### Definition and handling of treatment as usual

TAU is a heterogeneous comparator in trials of psychological interventions, as its content, intensity and availability may vary substantially across studies, healthcare systems and countries. This heterogeneity poses a potential threat to the transitivity assumption in network meta-analysis. To ensure sufficient comparability of TAU arms within the present IPD-NMA, we will code trial arms as TAU only if the following criteria are met: (1) participants allocated to TAU did not systematically receive any additional psychological intervention or structured psychobehavioural support provided as part of the trial protocol, and (2) participants did not receive, nor were informed that they would receive, access to the experimental intervention after a predefined waiting period.

If the criterion (1) is violated, the respective arm will be classified as *enhanced TAU* or allocated to another appropriate treatment node, depending on the nature of the additional intervention. Conditions that violate criterion (2) will be classified as waitlist control conditions, as expectancy effects associated with delayed access to the intervention may substantially influence outcomes. These distinctions will be explicitly considered when constructing the treatment network and when assessing the plausibility of the transitivity assumption.

### Analysis of subgroups or subsets

We will use the IPD dataset to investigate differential treatment effects across participant characteristics, such as baseline scores of the outcome variable and a history of early trauma, by pooling the stratified treatment effect modification coefficients of relevant predictors.

### Patient and public involvement

A lived experience advisory group will be consulted to provide input on the interpretation of the study findings and the development of dissemination materials. The results will be shared in accessible formats, including a lay summary co-produced with individuals with lived experience, to ensure findings are communicated effectively to patients and the wider public.

## Discussion

This IPD meta-analysis will address a critical yet often underexplored aspect of psychotherapeutic treatment: the occurrence of negative effects, including symptom deterioration, suicidality, AEs and dropout. While MBCT and CBASP are established interventions for DTD, potential harms remain insufficiently studied. By pooling individual-level data across trials, this study will provide a more precise and nuanced understanding of the prevalence, severity and predictors of negative outcomes in these therapies.

One of the strengths of this study will be the use of IPD, which enables detailed subgroup analyses and the examination of individual-level moderators. Inclusion of participant-level prognostic factors may also help to improve the plausibility of the transitivity assumption, which is crucial for the interpretability of NMA models—this is an advantage of analysing IPD versus aggregate data alone. However, there are potential limitations, including the variability in study designs and the number and/or quality of the included trials. Additionally, the transformation of different outcome measures into a common metric may result in some loss of information and/or measurement error. Despite these limitations, the findings of this analysis have the potential to inform both clinical decision-making and the development of guidelines that better balance benefit and risk. A clearer understanding of which patients are most vulnerable to negative effects may also support more personalised treatment approaches and enhance informed consent procedures.

### Ethics and dissemination

No local ethical review was necessary following consultation with the Swedish Ethical Review Authority. The investigators of the primary trials have obtained local ethical approval for sharing their data. TB and MN will oversee the database, and data analyses will be conducted by MH. Patient privacy will be ensured by adhering to the Karolinska Institutet guidelines on data management and storage. Data exchange is governed by inter-institutional data sharing agreements, and all data were pseudonymised before sharing. The data set will not be open access, but access may be shared on reasonable request.

The results of this meta-analysis will be disseminated through peer-reviewed journals, lay summaries and discussions with patient and lived experience groups. Additional dissemination methods will include conference presentations and social media announcements. By making the findings accessible to various stakeholders, we aim to enhance the understanding and implementation of CBASP and MBCT in treating chronic depression and DTD.

## Supplementary material

10.1136/bmjopen-2025-106362 online supplemental file 1
