# Supplementary material for: Negative effects of mindfulness-based cognitive therapy and cognitive behavioural analysis system of psychotherapy for patients with ‘difficult to treat’ depression: protocol for a systematic review and individual participant data meta-analysis
Source: BMJ Open. 2026 Apr 16;16(4):e106362. doi: 10.1136/bmjopen-2025-106362 (PMC13110557; doi:10.1136/bmjopen-2025-106362)
Supplement: online supplemental file 1 [file bmjopen-16-4-s001.pdf]

Appendix 1:

Search Terms for CBASP

Database	Search Term
Web of Science	(“MDE” OR “major depressive episode” OR “MDD” OR “major depressive disorder” OR “major depression” OR “depression” OR “dysthymia” OR “dysthymic”) AND (“chronic” OR “persistent” OR “treatment-resistant” OR “non-responders” OR “non-response” OR “non-remitted” OR “dysthymia” OR “dysthymic”) AND (“CBASP” OR “Cognitive behavioral analysis system of psychotherapy” OR “Cognitive-behavioral analysis system of psychotherapy”) AND (“randomized controlled trial” OR “randomised controlled trial” OR “randomized-controlled trial” OR “randomised-controlled trial” OR “RCT” OR “randomized” or “randomised” OR “randomly allocated” OR “randomly assigned”)
Scopus	(“MDE” OR “major depressive episode” OR “MDD” OR “major depressive disorder” OR “major depression” OR “depression” OR “dysthymia” OR “dysthymic”) AND (“chronic” OR “persistent” OR “treatment-resistant” OR “non-responders” OR “non-response” OR “non-remitted” OR “dysthymia” OR “dysthymic”) AND (“CBASP” OR “Cognitive behavioral analysis system of psychotherapy” OR “Cognitive-behavioral analysis system of psychotherapy”) AND (“randomized controlled trial” OR “randomised controlled trial” OR “randomized-controlled trial” OR “randomised-controlled trial” OR “RCT” OR “randomized” or “randomised” OR “randomly allocated” OR “randomly assigned”)
PubMed	(“MDE” OR “major depressive episode” OR “MDD” OR “major depressive disorder” OR “major depression” OR “depression” OR “dysthymia” OR “dysthymic”) AND (“chronic” OR “persistent” OR “treatment-resistant” OR “non-responders” OR “non-response” OR “non-remitted” OR “dysthymia” OR “dysthymic”) AND (“CBASP” OR “Cognitive behavioral analysis system of psychotherapy” OR “Cognitive-behavioral analysis system of psychotherapy”) AND (“randomized controlled trial” OR “randomised controlled trial” OR “randomized-controlled trial” OR “randomised-controlled trial” OR “RCT” OR “randomized” or “randomised” OR “randomly allocated” OR “randomly assigned”)
PsycInfo	(“MDE” OR “major depressive episode” OR “MDD” OR “major depressive disorder” OR “major depression” OR “depression” OR “dysthymia” OR “dysthymic”) AND (“chronic” OR “persistent” OR “treatment-resistant” OR “non-responders” OR “non-response” OR “non-remitted” OR “dysthymia” OR “dysthymic”) AND (“CBASP” OR “Cognitive behavioral analysis system of psychotherapy” OR “Cognitive-behavioral analysis system of psychotherapy”) **AND** (“randomized controlled trial” OR “randomised controlled trial” OR

	“randomized-controlled trial” OR “randomised-controlled trial” OR “RCT” OR “randomized” or “randomised” OR “randomly allocated” OR “randomly assigned”)
Embase	(‘MDE’ OR ‘major depressive episode’ OR ‘MDD’ OR ‘major depressive disorder’ OR ‘major depression’ OR ‘depression’ OR ‘dysthymia’ OR ‘dysthymic’) AND (‘chronic’ OR ‘persistent’ OR ‘treatment-resistant’ OR ‘non-responders’ OR ‘non-response’ OR ‘non-remitted’ OR ‘dysthymia’ OR ‘dysthymic’) AND (‘CBASP’ OR ‘Cognitive behavioral analysis system of psychotherapy’ OR ‘Cognitive-behavioral analysis system of psychotherapy’) AND (‘randomized controlled trial’ OR ‘randomised controlled trial’ OR ‘randomized-controlled trial’ OR ‘randomised-controlled trial’ OR ‘RCT’ OR ‘randomized’ or ‘randomised’ OR ‘randomly allocated’ OR ‘randomly assigned’)
Cochrane Controlled Trials	(“MDE” OR “major depressive episode” OR “MDD” OR “major depressive disorder” OR “major depression” OR “depression” OR “dysthymia” OR “dysthymic”) AND (“chronic” OR “persistent” OR “treatment-resistant” OR “non-responders” OR “non-response” OR “non-remitted” OR “dysthymia” OR “dysthymic”) AND (“CBASP” OR “Cognitive behavioral analysis system of psychotherapy” OR “Cognitive-behavioral analysis system of psychotherapy”) AND (“randomized controlled trial” OR “randomised controlled trial” OR “randomized-controlled trial” OR “randomised-controlled trial” OR “RCT” OR “randomized” or “randomised” OR “randomly allocated” OR “randomly assigned”)
Total	After duplicates removed

Search Terms for MBCT

Database	Search Term
Web of Science	(“MDE” OR “major depressive episode” OR “MDD” OR “major depressive disorder” OR “major depression” OR “depression” OR “dysthymia” OR “dysthymic”) AND (“chronic” OR “persistent” OR “treatment-resistant” OR “non-responders” OR “non-response” OR “non-remitted” OR “dysthymia” OR “dysthymic”) AND (“MBCT” OR "mindfulness-based cognitive therapy") AND (“randomized controlled trial” OR “randomised controlled trial” OR “randomized-controlled trial” OR “randomised-controlled trial” OR “RCT” OR “randomized” or “randomised” OR “randomly allocated” OR “randomly assigned”)
Scopus	(“MDE” OR “major depressive episode” OR “MDD” OR “major depressive disorder” OR “major depression” OR “depression” OR “dysthymia” OR “dysthymic”) AND (“chronic” OR “persistent” OR “treatment-resistant” OR “non-responders” OR “non-response” OR “non-remitted” OR “dysthymia” OR “dysthymic”) AND (“MBCT”

	OR "mindfulness-based cognitive therapy") AND (“randomized controlled trial” OR “randomised controlled trial” OR “randomized-controlled trial” OR “randomised-controlled trial” OR “RCT” OR “randomized” or “randomised” OR “randomly allocated” OR “randomly assigned”)
PubMed	(“MDE” OR “major depressive episode” OR “MDD” OR “major depressive disorder” OR “major depression” OR “depression” OR “dysthymia” OR “dysthymic”) AND (“chronic” OR “persistent” OR “treatment-resistant” OR “non-responders” OR “non-response” OR “non-remitted” OR “dysthymia” OR “dysthymic”) AND (“MBCT” OR "mindfulness-based cognitive therapy") AND (“randomized controlled trial” OR “randomised controlled trial” OR “randomized-controlled trial” OR “randomised-controlled trial” OR “RCT” OR “randomized” or “randomised” OR “randomly allocated” OR “randomly assigned”)
PsycInfo	(“MDE” OR “major depressive episode” OR “MDD” OR “major depressive disorder” OR “major depression” OR “depression” OR “dysthymia” OR “dysthymic”) **AND** (“chronic” OR “persistent” OR “treatment-resistant” OR “non-responders” OR “non-response” OR “non-remitted” OR “dysthymia” OR “dysthymic”) **AND** (“MBCT” OR "mindfulness-based cognitive therapy") **AND** (“randomized controlled trial” OR “randomised controlled trial” OR “randomized-controlled trial” OR “randomised-controlled trial” OR “RCT” OR “randomized” or “randomised” OR “randomly allocated” OR “randomly assigned”)
Embase	(‘MDE’ OR ‘major depressive episode’ OR ‘MDD’ OR ‘major depressive disorder’ OR ‘major depression’ OR ‘depression’ OR ‘dysthymia’ OR ‘dysthymic’) **AND** (‘chronic’ OR ‘persistent’ OR ‘treatment-resistant’ OR ‘non-responders’ OR ‘non-response’ OR ‘non-remitted’ OR ‘dysthymia’ OR ‘dysthymic’) **AND** (‘MBCT’ OR ‘mindfulness-based cognitive therapy’) **AND** (‘randomized controlled trial’ OR ‘randomised controlled trial’ OR ‘randomized-controlled trial’ OR ‘randomised-controlled trial’ OR ‘RCT’ OR ‘randomized’ or ‘randomised’ OR ‘randomly allocated’ OR ‘randomly assigned’)
Cochrane Controlled Trials	(“MDE” OR “major depressive episode” OR “MDD” OR “major depressive disorder” OR “major depression” OR “depression” OR “dysthymia” OR “dysthymic”) AND (“chronic” OR “persistent” OR “treatment-resistant” OR “non-responders” OR “non-response” OR “non-remitted” OR “dysthymia” OR “dysthymic”) AND (“MBCT” OR "mindfulness-based cognitive therapy") AND (“randomized controlled trial” OR “randomised controlled trial” OR “randomized-controlled trial” OR “randomised-controlled trial” OR “RCT” OR “randomized” or “randomised” OR “randomly allocated” OR “randomly assigned”)
