# Supplementary material for: Negative effects of mindfulness-based cognitive therapy and cognitive behavioural analysis system of psychotherapy for patients with ‘difficult to treat’ depression: protocol for a systematic review and individual participant data meta-analysis
Source: BMJ Open. 2026 Apr 16;16(4):e106362. doi: 10.1136/bmjopen-2025-106362 (PMC13110557; doi:10.1136/bmjopen-2025-106362)
Supplement: Supplementary file 2 [file bmjopen-2025-106362.reviewer_comments.pdf]

PEER REVIEW HISTORY

BMJ Open publishes all reviews undertaken for accepted manuscripts. Reviewers are asked to complete a checklist review form and are provided with free text boxes to elaborate on their assessment. These free text comments are reproduced below.

ARTICLE DETAILS

Title (Provisional)

Negative effects of Mindfulness-Based Cognitive Therapy and Cognitive Behavioral Analysis System of Psychotherapy for patients with ‘difficult to treat’ depression: protocol for a systematic review and individual participant data meta-analysis

Authors

Niemi, Maria; Michalak, Johannes; Velana, Maria; Barnhofer, Thorsten; Harrer, Mathias

---

VERSION 1 - REVIEW

**Reviewer**	**1**
**Name**	**Cohen, Sem**
**Affiliation**	**Amsterdam UMC Locatie AMC, Psychiatry**
**Date**	**02-Dec-2025**
**COI**	**None**

This is an important topic as the downsides of psychotherapy are an under researched topic. The authors are planning a thorough SR and IPDNMA which is laudable. I went through the protocol and found some relevant issues that I would want to raise in order to further improve the manuscript.

Introduction

There is a lot of attention given to TRD in the introduction, but it’s not in the inclusion criteria (Methods- Population) ? If TRD is not in the inclusion criteria, please remove from the introduction and the study objectives. It might be better to focus on the term chronic depression. For instance, what should be the definition of chronic? And, if PLEs are involved, should chronic still be a term that is used (my personal opinion is that the term has the downside of increasing learned helplessness and is stigmatising, and should therefore be replaced).

There seems to be a typo or some missing words in study objective #2.

See also my first point in ‘methods’, but the choice for MBCT and CBASP is quite specific. This does not have to be an issue, but is would be good to address this. Might a goal of the study

be to specifically compare MBCT to the most-studied intervention for chronic depression (namely CBASP) ? In that case, it would be important to clearly and transparently describe this, and add the study hypotheses. The issue of transparency in hypothesis testing is especially relevant as three of the authors are involved in MBCT development which has me believe they should have an a-priori idea on the matter.

Methods

Regarding the PICO (Intervention) :

Why ‘only’ CBASP and MBCT - they might be common for chronic depression but are not the only possible interventions? It might be fruitful to further elaborate on the choice for specifically these two. This could be done in the introduction - see remark there.

Regarding the PICO (Comparator/control) :

- The choice of comparators is quite limited. An upside of the NMA approach is that you can use indirect evidence - why not also include SSRIs and/or other psychotherapies? Or does that also fall under the umbrella of TAU?

- On that issue, it would be beneficial to further specify TAU: there should be attention to if the TAU is similar enough to be considered one node in the network. This also refers to my point regarding the transitivity assumption that should be tested.

Regarding the PICO (Outcome) :

- “Common metric scores will be converted into PHQ-9.” Now I’m not familiar with the practice of converting scales into the PHQ - could the authors specify how they will convert? Are there tables from studies that have attempted this? And how successful has this been? Adding this into the protocol would enhance transparency. This may be added for the MADRS, HAMD and other common scales.

- The authors will dichotomise the outcome for clinical interpretability. I do understand that ‘xx% of patients did deteriorate’ is more interpretable than for instance SMDs. But the downside is that power may be reduced by dichotomising the outcome, and information is lost. Why not use SMDs if the wish is to compare CBASP and MBCT? This can later be presented in the in a more clinically interpretable way in the manuscript.

- If dichotomisation is chosen by the authors (and defended), please specify how the cutoff of 6 points of the PHQ is found.

Regarding transitivity:

Please explain how you will examine transitivity assumption? Which patient data would you take as relevant for testing for transitivity, and based on which prior evidence? For instance, I think that current episode duration should be one important characteristic that should be checked beforehand. Of course, using an IPD approach stretches what you can do to safeguard transitivity, but it doesn’t altogether decrease the importance of transitivity.

Regarding regression analysis:

I would like to see a bit more extensive explanation on the reason for choosing the patient characteristics. Based on which literature, and/or clinical expertise did the authors come to this set of characteristics?

In data extraction:

- “If we are unable to get the IPD for a study.. etc” . This is a clear solution for a foreseeable problem - it may be good to stress that this data will not be able to be used in the regression analysis for patient-level characteristics. Transitivity should be strictly guarded in this group.

- Adverse events are mentioned but not well-defined: please specify what you will and will not consider an AE, especially as the reporting/specification may be inconsistent in psychotherapy trials.

- On that matter, have the authors considered adding acceptability (dropout) as outcome? If there are less negative effects, but the dropout is higher in one arm, this is relevant information. Using imputation with missing outcome data may have the downside of concealing this issue.

Regarding data analysis:

- Flat normal/weakly informative priors - how will you systematically decide which to choose?

- For missing data which data could you hypothesise to be not missing at random. How wil you account for data NMAR?

Ethics and dissemination

Some of the wording is in past tense which gives the impression that the data has already been shared. Please specify if this is true (meaning that the search has already been finalised and the data has been centralised).

VERSION 1 - AUTHOR RESPONSE

Reviewer: 1
Dr. Sem Cohen, Amsterdam UMC Locatie AMC
Comments to the Author:
This is an important topic as the downsides of psychotherapy are an under researched topic. The authors are planning a thorough SR and IPDNMA which is laudable. I went through the protocol and found some relevant issues that I would want to raise in order to further improve the manuscript.

We thank the reviewer for the positive evaluation of the importance of this topic and for acknowledging the planned systematic review and IPD network meta-analysis.

Introduction

We have substantially revised the Introduction. From the beginning the revised version more strongly focuses on negative effects of MBCT. This revision was undertaken to more clearly align the background with the primary aims of the study and to provide a transparent and coherent rationale for the choice of CBASP as a comparator, particularly in the context of evaluating negative effects in a vulnerable population with difficult-to-treat depression.

There is a lot of attention given to TRD in the introduction, but it's not in the inclusion criteria (Methods- Population) ? If TRD is not in the inclusion criteria, please remove from the introduction and the study objectives. It might be better to focus on the term chronic depression. For instance, what should be the definition of chronic? And, if PLEs are involved, should chronic still be a term that is used (my personal opinion is that the term has the downside of increasing learned helplessness and is stigmatising, and should therefore be replaced).

We thank the reviewer for this helpful comment. We agree that the previous emphasis on treatment-resistant depression (TRD) was not sufficiently aligned with the inclusion criteria. In response, we have removed TRD from the introduction and study objectives and consistently adopted the concept of difficult-to-treat depression (DTD) throughout the manuscript.
DTD is a broader and more inclusive construct that captures persistent and impairing depressive courses without relying on narrow or heterogeneous definitions of treatment resistance. DTD will serve as umbrella term accommodate the whole range of studies investigated under operational definitions of non-response/non-remission, treatment resistance and chronic depression. We deliberately chose DTD over the term chronic depression, as definitions of chronicity vary considerably across studies and the term may carry stigmatizing connotations and risk reinforcing learned helplessness, particularly in vulnerable populations. However, as it has been frequently used in study criteria, it will need to feature in our operational definitions.
We have revised the Introduction and Methods accordingly to ensure conceptual clarity and full consistency with the inclusion criteria.

We have rewritten the paragraph in the Introduction accordingly:

Individuals with difficult-to-treat depression (DTD)—a broader and clinically meaningful construct encompassing persistent, impairing depressive courses regardless of strict treatment resistance criteria—represent a population of particular relevance for the study of negative effects. DTD is associated with elevated vulnerability, including higher rates of early life adversity and suicidality (REF), factors that have also been linked to increased risk of adverse experiences during psychological interventions (REF).

We have rewritten the Participants section accordingly:

"We will include studies recruiting adults with a current major depressive disorder (MDD) as well as trials enrolling mixed samples with either residual depressive symptoms or a current depressive episode in the context of a persistent, chronic, or difficult-to-treat course. In trials including such mixed samples, participants presenting with residual symptoms only will be excluded from the primary one-stage IPD analyses. As DTD does not rely on a single standardized operational definition, we will apply a broad and inclusive approach and include studies that defined eligibility based on prior non-response or non-remission, treatment resistance, and/or chronicity. Studies including participants younger than 18 years, and studies exclusively recruiting participants older than 65 years, will be excluded."

There seems to be a typo or some missing words in study objective #2.

We have addressed the typo.

See also my first point in 'methods', but the choice for MBCT and CBASP is quite specific. This does not have to be an issue, but is would be good to address this. Might a goal of the study be to specifically compare MBCT to the most-studied intervention for chronic depression (namely CBASP) ? In that case, it would be important to clearly and transparently describe this, and add the study hypotheses. The issue of transparency in hypothesis testing is especially relevant as three of the authors are involved in MBCT development which has me believe they should have an a-priori idea on the matter.

We more explicitly addressed the choice CBASP as a comparator:

"In order to study the negative outcomes of MBIs, it is therefore timely to compare these to other established treatment methods among this patient population. The Cognitive Behavioral Analysis System of Psychotherapy (CBASP, REF) is an empirically supported treatment specifically developed for individuals with persistent depression and has been evaluated in randomized controlled trials targeting this population (REF). Because CBASP is the most-studied intervention for DTD (i.e., persistent depression), it represents a clinically meaningful and appropriate comparator for MBCT in patients with DTD.
Notably, while both interventions are applied in populations characterized by persistent depressive symptoms, they differ fundamentally in their therapeutic focus and proposed mechanisms of change. CBASP emphasizes interpersonal learning and perceived interpersonal consequences, whereas MBCT primarily targets decentering from maladaptive cognitive and emotional processes through mindfulness practice. Moreover, MBCT is typically delivered in a group format, whereas CBASP is most commonly delivered as an individual, one-to-one psychotherapy.
Di."

With regard to hypotheses, we would like to emphasize that the present IPD meta-analysis is explicitly exploratory with respect to the comparative rates of negative effects. Given the limited and heterogeneous evidence on negative effects of both MBCT and CBASP, and the lack of direct comparative data, we did not formulate a priori directional hypotheses regarding differences in deterioration, suicidality, adverse events, or dropout.
We acknowledge that several authors have been involved in the development and evaluation of MBCT. For this reason, we considered it particularly important to avoid prespecified assumptions favoring either intervention and to transparently frame the analyses as hypothesis-generating rather than confirmatory. This exploratory approach is now explicitly stated in the Objectives sections of the revised manuscript:

"Given the absence of prior direct comparative evidence all comparative analyses were specified as exploratory and non-directional."

Methods
Regarding the PICO (Intervention) :
Why 'only' CBASP and MBCT - they might be common for chronic depression but are not the only possible interventions? It might be fruitful to further elaborate on the choice for specifically these two. This could be done in the introduction - see remark there.

We addressed this in the Introduction.

Regarding the PICO (Comparator/control) :
- The choice of comparators is quite limited. An upside of the NMA approach is that you can use indirect evidence - why not also include SSRIs and/or other psychotherapies? Or does that also fall under the umbrella of TAU?

We agree that, in principle, a network meta-analytic approach allows the inclusion of multiple comparators and the use of indirect evidence. However, in the present study, the restriction of comparators was a deliberate and conceptually motivated choice.
The primary aim of this IPD meta-analysis is not to provide a comprehensive ranking of treatments, but to conduct a focused and clinically meaningful comparison of negative effects between MBCT and CBASP in patients with difficult-to-treat depression. CBASP was selected, as stated above, because it is the most extensively studied psychotherapy specifically developed for this population and therefore represents a particularly relevant reference treatment.
Instead of analyzing antidepressant medication (ADM) as a specific node in our network, we will analyze it as a moderator. This allows us to use information on ADM also in RCTS in which ADM was not a treatment arm but was given to some patients as treatment as usual TAU.
We have clarified this rationale in the revised manuscript and now explicitly state that the network structure is intentionally restricted in order to maximize clinical interpretability and methodological coherence when examining negative effects in a vulnerable population.

We included the following sentence in the manuscript:

“The analytic focus is on MBCT and CBASP as the primary interventions of interest, enabling a clinically interpretable comparison of safety and acceptability within a relatively homogeneous population and intervention context. Control conditions are incorporated as comparators within the network to allow estimation of relative effects between MBCT and CBASP through integration of direct and indirect evidence.”

- On that issue, it would be beneficial to further specify TAU: there should be attention to if the TAU is similar enough to be considered one node in the network. This also refers to my point regarding the transitivity assumption that should be tested.

We agree that the comparability of care as usual arms is a common issue in meta-analyses of psychological interventions, since (i) the definition of TAU can differ strongly across trials, (ii) “usual care” can be inherently different in quality and quantity across countries and world regions, and (iii) because uptake of treatment in CAU comparisons is often not recorded. In this IPD-NMA, we will only code trial arms as TAU if (1) allocated patients did not systematically receive any additional type of psychological intervention or psychobehavioral support provided systematically as part of the trial protocol, and (2) if patients did not receive, or were told that they will receive, access to the studied intervention after a waiting period. If a trial violates (1), we will consider adding “enhanced TAU” or another suitable treatment node to the network. Conditions that do not align with (2) will be consider waitlist-controls, since this can great influence effects.

We now further clarify this classification in the manuscript. We agree with the reviewer that this point also touches on the point of network transitivity assumption. This point is addressed further below.

“Definition and handling of treatment as usual (TAU)
Treatment as usual (TAU) is a heterogeneous comparator in trials of psychological

interventions, as its content, intensity, and availability may vary substantially across studies, health care systems, and countries. This heterogeneity poses a potential threat to the transitivity assumption in network meta-analysis. To ensure sufficient comparability of TAU arms within the present IPD-NMA, we will code trial arms as TAU only if the following criteria are met: (1) participants allocated to TAU did not systematically receive any additional psychological intervention or structured psychobehavioral support provided as part of the trial protocol, and (2) participants did not receive, nor were informed that they would receive, access to the experimental intervention after a predefined waiting period.
If criterion (1) is violated, the respective arm will be classified as enhanced TAU or allocated to another appropriate treatment node, depending on the nature of the additional intervention. Conditions that violate criterion (2) will be classified as waitlist control conditions, as expectancy effects associated with delayed access to the intervention may substantially influence outcomes. These distinctions will be explicitly considered when constructing the treatment network and when assessing the plausibility of the transitivity assumption."

Regarding the PICO (Outcome) :
- "Common metric scores will be converted into PHQ-9." Now I'm not familiar with the practice of converting scales into the PHQ - could the authors specify how they will convert? Are there tables from studies that have attempted this? And how successful has this been? Adding this into the protocol would enhance transparency. This may be added for the MADRS, HAMD and other common scales.

We give more information on the common metrics approach in the manuscript including references:

"To ensure comparability of symptom severity scores assessed with different instruments, values will be transformed to the PHQ-9 metric using a validated common metric approach (REF). The common metric approach transforms scores obtained from different, but conceptually comparable, outcome measures onto a single standardized scale, thereby enabling direct comparison and joint analysis of outcomes across studies. If a measure is not part of the common metric, it will be converted using validated conversion tables to derive the common metric (REF).

- The authors will dichotomise the outcome for clinical interpretability. I do understand that 'xx% of patients did deteriorate' is more interpretable than for instance SMDs. But the downside is that power may be reduced by dichotomising the outcome, and information is lost. Why not use SMDs if the wish is to compare CBASP and MBCT? This can later be presented in the in a more clinically interpretable way in the manuscript.

Dichotomization is commonly used in research on negative effects of psychotherapy (e.g. Cuijpers et al., 2018) because it facilitates the identification of clinically meaningful deterioration, which often represents relatively rare but clinically critical events. When the majority of patients improve, such events may be obscured in analyses based on standardized mean differences, which primarily reflect average treatment effects. As a result, SMDs may lack sensitivity to detect small subgroups experiencing clinically significant worsening.
Importantly, our primary aim is not to estimate overall efficacy differences between MBCT and CBASP, but to compare the incidence of negative outcomes, including deterioration. We therefore consider dichotomous indicators of clinically significant change to be the most appropriate and clinically interpretable operationalization for this research question.

We have added the following paragraph to the manuscript:

“We will create a dichotomous outcome indicating deterioration. Dichotomization is commonly used in research on negative effects of psychotherapy (e.g. REF) because it facilitates the identification of clinically meaningful deterioration, which often represents relatively rare but clinically critical events.
.”

- If dichotomisation is chosen by the authors (and defended), please specify how the cutoff of 6 points of the PHQ is found.

We will define clinically relevant deterioration on the PHQ using a threshold of a ≥6-point increase from baseline, based on prior work indicating that this cutoff exceeds measurement error and corresponds to a reliable and clinically meaningful worsening of depressive symptoms (see reference in the manuscript).

“According to PHQ-9 conventions, deterioration will be defined as an increase of 6 or more points (REF).”

Moreover, since the 6-point change threshold represents a conservative estimate, often leading to sparse data, we decided to add the following paragraph including an additional criterion:

“A definition of deterioration using the 6-point change threshold may lead to sparse data (e.g. one and “double-zero” cells), which are often problematic for meta-analytic pooling [28]. As a mitigation strategy, if zero cells occur in two or more trials, we will therefore also consider modeling deterioration as any negative change compared to baseline, so as to avoid computational problems.

Regarding transitivity:
Please explain how you will examine transitivity assumption? Which patient data would you take as relevant for testing for transitivity, and based on which prior evidence? For instance, I think that current episode duration should be one important characteristic that should be checked beforehand. Of course, using an IPD approach stretches what you can do to safeguard transitivity, but it doesn’t altogether decrease the importance of transitivity.

We thank the reviewer for mentioning this important point. As mentioned by the reviewer, analyzing IPD comes with the added benefit that patient-level prognostic factors can be integrated into the model, which may help safeguard transitivity. We now describe in greater detail which patient-level variables will be adjusted as prognostic factors with the IPD-NMA, and under what constraints.

We now also provide greater detail on other measures to assess the plausibility of the transitivity assumption. For once, we will create graphical summaries of all relevant baseline indicators that will also be considered in treatment-covariate interaction analyses: age, gender, marital status, education, employment, childhood adversity, age at onset of depression, number of previous episodes, current episode duration, current suicidality, current antidepressant medication, past antidepressants and treatment-resistance to antidepressants, co-morbid anxiety disorder, comorbid obsessive-compulsive disorder, co-morbid post-traumatic stress disorder, co-morbid substance abuse or dependence, co-

morbid eating disorder, co-morbid personality disorder, and level of depressive symptoms at baseline. This will be used to detect potentials sets of comparisons with strongly diverging baseline risk. Quantitively, inconsistency will be assessed by rerunning a one-stage IPD-NMA inconsistency model, as well as a two-stage netsplit approach (Dias et al., 2010). We now specify these points in greater detail in the manuscript.

The presence of network consistency will be explored by several approaches. First, we will generate graphical summaries of baseline indicators within the IPD (e.g., box-whiskers plots grouped by comparison). This will be used to identify potentially systematic divergences in the baseline risk across comparisons within the network, which can be the cause of inconsistency. Quantitatively, the transitivity assumption will be assessed by (1) calculating the agreement between direct and indirect evidence for comparisons in an inconsistency model including an additional inconsistency parameter *w*, and (2) running a two-stage model using the “net split” approach (REF).

Regarding regression analysis:
I would like to see a bit more extensive explanation on the reason for choosing the patient characteristics. Based on which literature, and/or clinical expertise did the authors come to this set of characteristics?

We now provide relevant references to justify our selection of patient characteristics.

In data extraction:
- “If we are unable to get the IPD for a study.. etc” . This is a clear solution for a foreseeable problem - it may be good to stress that this data will not be able to be used in the regression analysis for patient-level characteristics. Transitivity should be strictly guarded in this group.

We agree that studies for which IPD cannot be obtained can only contribute to analyses based on study-level (aggregate) information. Therefore, if IPD are unavailable, extracted aggregate data (e.g., adverse events) will be used and these studies will not be included in patient-level regression or moderator analyses examining individual participant characteristics.
We have clarified this in the manuscript:

“If we are unable to get IPD for a study, we will extract aggregate data (e.g. on adverse events) from the paper of this study. Studies for which only aggregate data are available cannot be included in patient-level regression analyses examining individual participant characteristics. We will also carefully evaluate the transitivity assumption for analyses including aggregate-only studies.”

- Adverse events are mentioned but not well-defined: please specify what you will and will not consider an AE, especially as the reporting/specification may be inconsistent in psychotherapy trials.

Thank you for highlighting this. We agree that adverse events (AEs) are inconsistently defined and reported across psychotherapy trials, which limits comparability and makes quantitative synthesis problematic.
To ensure methodological transparency and avoid misclassification, we will therefore not pool AEs as secondary quantitative outcome in the IPD meta-analysis or patient-level regression analyses. Instead, we will extract and report, for each included study, (i) whether and how AEs were assessed, and (ii) the number and type of reported AEs (and serious

AEs, if available). This information will be presented descriptively in a dedicated table and summarized narratively.

“Reporting and definitions of adverse events (AEs) vary substantially across psychotherapy trials and are often not assessed using standardized procedures. For this reason, AEs will not be synthesized as a quantitative outcome in the primary IPD meta-analysis or included in patient-level moderator analyses. Instead, we will extract and present, for each study, the study-specific method of AE assessment and the number and type of reported AEs (and serious AEs, where available). These data will be summarized descriptively in a dedicated table.”

In addition we decided to make some specification how to analyze suicidality:

“Suicidality will be analyzed as one of the secondary outcomes and will be harmonized across studies using a predefined hierarchical approach. When available, data on suicidality will be obtained from clinician-rated items (e.g., Hamilton Depression Rating Scale, Item 3) and self-report items (e.g., BDI, Item 9). If multiple measures of suicidality are used in a single study, preference will be given to the measure most commonly used across studies. Dichotomous outcomes will be derived to indicate the presence or emergence of suicidality. Participants will be classified as having suicidality if they scored ≥ 1 on the respective suicidality item. *Emergent suicidality* will be defined as any occurrence of suicidality among participants without indication of suicidality at baseline, and *worsening of suicidality* as an increase of ≥ 1 point from baseline among participants who already report some level of suicidality.”

- On that matter, have the authors considered adding acceptability (dropout) as outcome? If there are less negative effects, but the dropout is higher in one arm, this is relevant information. Using imputation with missing outcome data may have the downside of concealing this issue.

We agree that treatment dropout is a relevant indicator of acceptability and potential burden, particularly when interpreting negative effects. In response, we have explicitly included dropout as a secondary outcome and describe it as an additional indicator of treatment acceptability and potential burden.
We also agree that missing outcome data and imputation could conceal differences in acceptability if not handled transparently. Therefore, dropout will be analysed separately as an outcome in its own right, and we will report attrition rates by treatment arm across studies. Multiple imputation will be applied to symptom outcomes where appropriate, but it will not replace the explicit reporting and comparison of dropout between conditions. We added the following sentences in the introduction and method section:

“Treatment dropout constitutes an additional indicator of treatment acceptability and potential burden, and may reflect negative experiences or tolerability problems during treatment.”

„We will report and compare dropout rates between arms as a distinct acceptability outcome; this will complement the handling of missing symptom data via multiple imputation.“

Regarding data analysis:
- Flat normal/weakly informative priors - how will you systematically decide which to choose?

We now provide a more detailed description of the priors that will be used in the Bayesian analyses, and elaborate more on why they were chosen. One major choice prior in Bayesian

(N)MA concerns the between-study heterogeneity variance, where weakly informative priors are typically recommended (see e.g. Harrer et al., 2021, chap. 13). In line with previous recommendations for log-odds models, a weakly informative Half-Normal (0,0.05) prior will be used, since it centers the prior mass around generally sensible heterogeneity values, and has several computational benefits (Röver et al., 2021).

Since our outcome (deterioration) will likely be sparse, weakly informative priors will also be used for trial intercepts and slopes/predictors. This is in line with recommendations by Gelman and colleagues (2013; chap. 16.3) to avoid issues with complete separation and related problems in sparse outcome data. As recommended by Gelman et al., we will also employ Cauchy/diffuse t-priors, with exact specifications now provided in the manuscript. As a sensitivity analyses, if feasible, we will also rerun the model with flat normal priors.

"The primary outcome, symptom deterioration, will be modeled using a binomial logit-link. Let y_{ik} be the deterioration status of some participant *i* included in study *k* (with *k*=1, …, *K* studies included the meta-analysis), where *j*=*b* represents the baseline arm, and *j*=*t* the intervention arm in *k*. The one-stage IPD-NMA model will then be defined as:

[Formula omitted]

where ***d*** represents a vector containing the effect sizes in comparison to the reference treatment implicated by our network, and with **Σ** being a heterogeneity variance-covariance matrix with off-diagonal elements $\rho\tau^2$, where ρ is set to a non-zero value to account for multi-arm studies. A weakly informative Half-Normal(0,0.5) prior will be used by default square root of the heterogeneity variance, τ (REF). Given that we expect deterioration to be fairly uncommon, to avoid issues with sparse data, weakly informative Cauchy(0,2.5) and Cauchy(0,10) will be used for all slopes and intercepts, respectively (REF). Analyses with flat $\mathcal{N}(0,100^2)$ priors will be conducted as a sensitivity analysis if feasible."

Harrer, M., Cuijpers, P., Furukawa, T.A., & Ebert, D.D. (2021). *Doing Meta-Analysis with R: A Hands-On Guide*. Boca Raton, FL and London: Chapman & Hall/CRC Press. ISBN 978-0-367-61007-4.

Röver, C., Bender, R., Dias, S., Schmid, C. H., Schmidli, H., Sturtz, S., ... & Friede, T. (2021). On weakly informative prior distributions for the heterogeneity parameter in Bayesian random-effects meta-analysis. Research Synthesis Methods, 12(4), 448-474.

- For missing data which data could you hypothesise to be not missing at random. How wil you account for data NMAR?

We agree with the reviewer that consideration of different missingness mechanisms is relevant. Multiple imputation is used as the default analytic approach, which operates under MAR. We now also prespecify an additional sensitivity analysis in which models are rerun after imputing missing values under a non-ignorable MNAR pattern, in which we assume that dropouts are associated with (reliable) deterioration.

. Missing data will be handled using multilevel multiple imputation with *m*=50 sets. All models will be fitted within multiply imputed data, and parameter estimates will be aggregated by mixing the posterior draws. Multiple imputation operates under the missing at random (MAR) assumption. As a sensitivity analysis, we will therefore also rerun the main model under the "worst-case" scenario that all individuals who were lost to follow-up experienced reliable symptom deterioration.

.

Ethics and dissemination
Some of the wording is in past tense which gives the impression that the data has already been shared. Please specify if this is true (meaning that the search has already been finalised and the data has been centralised).

Thank you for this comment. We agree that some wording in the manuscript could be interpreted as implying that data sharing and centralization had already been completed. We have revised the text to consistently use future tense where appropriate and to clarify the current project status at the time of submission.

“The study was initiated in February 2023, including study planning and preparatory work as well as the initiation of IPD requests. The systematic literature search was conducted up to May 30, 2025, in accordance with the registered protocol, and the study is expected to be completed by the end of 2026.”

VERSION 2 - REVIEW

**Reviewer**	**1**
**Name**	**Cohen, Sem**
**Affiliation**	**Amsterdam UMC Locatie AMC, Psychiatry**
**Date**	**30-Jan-2026**
**COI**	

I thank the authors for their clear and extensive answers, and the diligent subsequent revisions in the protocol. I have no further comments and wish them good luck in conducting the analyses/ writing the paper.
